# Supplementary material for: Association between oral microbiome and five types of respiratory infections: a two-sample Mendelian randomization study in east Asian population
Source: Front Microbiol. 2024 Apr 10;15:1392473. doi: 10.3389/fmicb.2024.1392473 (PMC11039966; doi:10.3389/fmicb.2024.1392473)
Supplement: Supplementary file 1 [file Data_Sheet_1.zip › Supplementary Materials/MR plots of saliva/Tonsillitis/s__Porphyromonas_endodontalis_mgs_3068/forest.pdf]

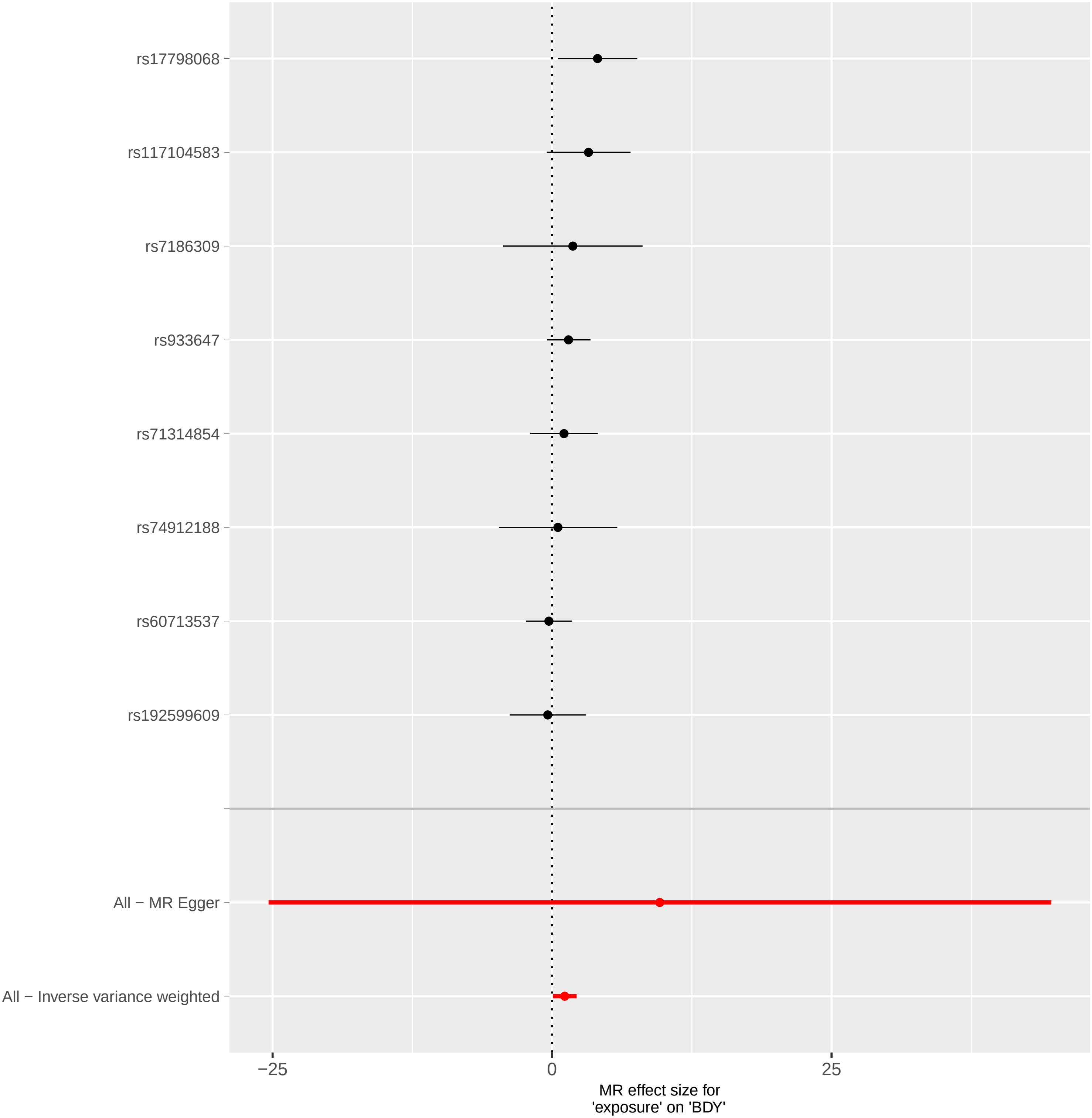
rs17798068
rs117104583
rs7186309
rs933647
rs71314854
rs74912188
rs60713537
rs192599609
All − MR Egger
All − Inverse variance weighted
−25
0
25
MR effect size for
'exposure' on 'BDY'
